# In Silico Structural Analysis Predicting the Pathogenicity of PLP1 Mutations in Multiple Sclerosis

**DOI:** 10.3390/brainsci13010042

**Published:** 2022-12-24

**Authors:** Antigoni Avramouli, Marios G. Krokidis, Themis P. Exarchos, Panagiotis Vlamos

**Affiliations:** Bioinformatics and Human Electrophysiology Laboratory, Department of Informatics, Ionian University, 491 00 Corfu, Greece

**Keywords:** myelin proteolipid protein, protein structure prediction, functional analysis, multiple sclerosis

## Abstract

The X chromosome gene *PLP1* encodes myelin proteolipid protein (PLP), the most prevalent protein in the myelin sheath surrounding the central nervous system. X-linked dysmyelinating disorders such as Pelizaeus–Merzbacher disease (PMD) or spastic paraplegia type 2 (SPG2) are typically caused by point mutations in *PLP1*. Nevertheless, numerous case reports have shown individuals with *PLP1* missense point mutations which also presented clinical symptoms and indications that were consistent with the diagnostic criteria of multiple sclerosis (MS), a disabling disease of the brain and spinal cord with no current cure. Computational structural biology methods were used to assess the impact of these mutations on the stability and flexibility of PLP structure in order to determine the role of *PLP1* mutations in MS pathogenicity. The analysis showed that most of the variants can alter the functionality of the protein structure such as R137W variants which results in loss of helix and H140Y which alters the ordered protein interface. In silico genomic methods were also performed to predict the significance of these mutations associated with impairments in protein functionality and could suggest a better definition for therapeutic strategies and clinical application in MS patients.

## 1. Introduction

Myelination is an important process of the CNS that provides electrical insulation to axons and facilitates the transmission of nerve impulses. This protective layer is formed by Schwann cells in the peripheral nervous system, while oligodendrocytes form the sheath in the CNS [1]. The myelin sheath is a multi-layered membrane composed of proteins and lipids (approximately 30% and 70%, respectively). The lipid composition contains high amounts of cholesterol, phospholipids and glycolipids [2]. PLP is one of the major myelin proteins which, together with the DM20 isoform resulting from alternative splicing, constitutes 50% of the total protein. PLP plays a crucial role in the formation and maintenance of proper myelin structure and stability in the CNS [3]. It is a transmembrane and hydrophobic protein, with 48% of its sequence being non-polar or aromatic amino acids including 14 cysteine residues, which either undergo post-translational modifications and bind to fatty acids or are involved in intramolecular disulfide bonds. It has been observed that patients suffering from MS have an increased population of T-cells specific for PLP peptides and increased levels of anti- PLP_181–230_ specific antibodies were found in serum levels compared to healthy individuals and patients with other neurological diseases [4]. Human and rodent PLP share several epitopes that are recognized by T cells [5]. Other main counterparts are myelin basic protein (MBP), which constitutes 30% of the total myelin protein in the CNS, myelin oligodendrocyte glycoprotein (MOG) and myelin-associated glycoprotein (MAG) [6,7,8]. Smaller percentage is occupied by alpha-beta crystallin, a small heat shock protein [9]. 

Multiple sclerosis (MS) is a chronic demyelinating inflammatory condition affecting the human central nervous system (CNS) [10]. It is unclear whether MS begins in the periphery, through activation of immune cells that then penetrate the CNS and cause damage, or within the CNS through primary damage to myelin or oligodendrocyte [11,12]. This could be the result of mutations in molecules encoding essential myelin or oligodendrocyte components, even though genome-wide association studies have not indicated substantial associations with any of these in MS [13]. However, the possibility that mutations in genes encoding these components are present in some proportions of MS patients remains under consideration. To that end, missense mutations in *PLP1* gene have been described in patients with clinical symptoms consistent with an MS diagnosis, such as an amino acid substitution at residue 31 of PLP (L31V) in a female patient with primary progressive MS [14]. Mutations can largely affect protein functionality, hence analysis of potential alterations in protein tertiary structure can reveal new evidence for their effect on phenotype. A novel mutation in *PLP1* exon 2 that changed leucine to arginine (L31R) was reported in a mother and her son through sequencing of the *PLP1* gene [15]. In a recent study, a mother and daughter with a preliminary diagnosis of primary progressive MS carried a nonsense mutation at codon 210 T > G [16]. Moreover, a L31P mutation was also associated with severe PMD [17]. The transmembrane portion of PLP1/DM20 proteins could be disrupted by both mutations, affecting intracellular trafficking. Neuroinflammation and axonal neurodegeneration was also reported in mice carrying the R137W and L31R mutations by one year of age [18].

The structure and function of the native protein may be significantly altered by missense mutations, particularly those in the coding area that modify the amino acid configuration. In order to determine the impact of each nonsynonymous single nucleotide polymorphism (nsSNP) in a related protein, it is common practice to functionally compare mutant proteins with their wild-type counterparts associated with specific traits in vitro [19]. However, the experimental design for each mutational modification is time- and labor-intensive. Thus, it is feasible and cost-effective to perform data mining for mutational analysis and functional prediction on protein properties using computational methods [20]. The three-dimensional (3D) structure of a protein has a pivotal role in protein’s functional characterization. There are many efficient structural biology algorithms for predicting tertiary protein structures based on their amino acid sequences. Therefore, considering the role of the *PLP1* gene in spontaneous myelin and axonal damage, we retrieved all mutations in the *PLP1* gene related to MS. Using in silico structural and functional analyses, this study aimed to describe potential disease-associated variants of the *PLP1* gene. 

## 2. Materials and Methods

A variety of different computational approaches was used to screen out the functional effects of the variants in the *PLP1* gene related to MS. The methodology we followed is divided into four distinct levels, including (i) primary data collection, (ii) creation of the 3D protein structures, (iii) 3D protein structure comparison process (iv) variant functional analysis. A baseline method raised from pure bioinformatics approaches was utilized as a benchmark for validation. 

### 2.1. Summary of Variants

Five variants of *PLP1* gene and specifically the association of three of them with multiple sclerosis were analyzed in our study. The transcript sequence and the protein encoded by the *PLP1* gene were retrieved from the Ensembl database [21]. Then, the UniProt ID for the amino acid (P60201–MYPR_HUMAN) was obtained from UniProt Protein Database.

### 2.2. In Silico Methods for Predicting Mutation Significance

dbNSFP database was used for functional prediction and annotation of potential non-synonymous single-nucleotide variants (nsSNVs). The current version of this high-performance variant annotation tool can be queried to extract prediction scores from 38 algorithms [22,23]. dbNSFP also provides conservation scores and supplementary data, such as allele frequencies, functional gene descriptions, gene expression and gene interaction data, etc. MutPred2 machine learning-based approach was implemented to predict the pathogenicity of amino acid substitutions and their molecular mechanisms [24]. 

Further computational tools such as ANNOVAR (https://annovar.openbioinformatics.org/en/latest/, accessed on 16 September 2022), KGGSeq (http://pmglab.top/kggseq/, accessed on 16 September 2022), VarSome (https://varsome.com, accessed on 16 September 2022), UCSC Genome Browser’s Variant Annotation Integrator (http://genome.ucsc.edu/cgi-bin/hgVai, accessed on 19 September 2022), Ensembl Variant Effect Predictor (http://www.ensembl.org/info/docs/tools/vep/index.html, accessed on 20 September 2022), SnpSift (https://pcingola.github.io/SnpEff/, accessed on 20 September 2022) and HGMD (https://www.hgmd.cf.ac.uk/ac/index.php, accessed on 22 September 2022) were used to strengthen the analysis, and the outcomes were validated using each platform separately. The algorithms utilized in this study are publicly accessible for all academic, non-commercial uses. 

### 2.3. Protein Stability Correlation Analysis

The correlation between mutations and protein stability was analyzed based on a lesser decrease in free energy (ΔG or dG). Alterations in protein stability are determined by differences in free energy (ΔΔG or ddG) between wild-type and mutant proteins [25]. The DynaMut server was used to assess the effect of a single point mutation on protein stability, conformation, and flexibility, and to visualize protein dynamics [26]. DynaMut provides more accurate (*p*-value < 0.001) assessments of the effects of single mutations on protein stability than other well-established methods. In addition, the DynaMut server defines ΔΔG ≥ 0 as stabilizing and ΔΔG < 0 for comparison purposes. In addition, Site Directed Mutator (SDM) server was used to estimate the change in protein stability following mutation [27]. 

### 2.4. Analysis of Protein Structural Conformation and Conservation

ConSurf server (http://consurf.tau.ac.il/, accessed on 7 October 2022) was utilized to identify highly conserved functional areas of the PLP1 gene-encoded protein [28].

### 2.5. Prediction of the Secondary Structure

Using the PSIPRED server (http://bioinf.cs.ucl.ac.uk/psipred/, accessed on 10 October 2022), the secondary structure of PLP1 was predicted [29]. It is based on a two-stage neural network with position-specific scoring matrices derived from PSI-BLAST to predict the available secondary structures of a protein.

### 2.6. Homology Modeling

SWISS-MODEL was utilized to determine the three-dimensional structure of PLP1. The CAMEO system determines the precision of the generated model. SWISS-MODEL is based on evolutionary information and searches a high-throughput template library (SMTL) for the optimum sequence–template alignment to construct the model [30]. Phyre-2 server was used to predict the homology-based three-dimensional structure of the query amino acid sequence [31]. I-TASSER was selected for protein structure prediction and structure-based function annotation [32,33,34]. Initially, structural templates from the PDB are discovered using LOMETS, a multithreaded algorithm. With the templates as guides, full-length atomic models are then constructed using simulations of fragment assembly iterations. The 3D models are re-run through the BioLiP database of protein functions to gain insight into the target’s function. C-I-TASSER, an enhanced version of I-TASSER designed to accurately predict protein structures and functions was used to generate inter-residue contact maps beginning with a query sequence [34]. The structural templates of the PDB are derived by the multithreaded method LOMETS, and their full-length atomic models are constructed using contact maps and replica exchange Monte Carlo simulations. Finally, COFACTOR uses the structural model to deduce the protein’s biological functions. C-I-TASSER produces significantly more accurate models than I-TASSER in large-scale benchmark tests.

### 2.7. Mutated Structure Prediction

Once the mutations were identified, the construction protein-based structures in PDB format were followed. I-TASSER and DynaMut servers were used to perform the transformation of the amino acid sequences to 3D protein models. Then, for each model, structural alignment was carried out and the structural similarity score was calculated. The TM-align and TM-score algorithms were selected for the alignment and the similarity score calculation, respectively [35]. 

### 2.8. Protein Three-Dimensional Model Verification

Three-dimensional structures were validated using Ramachandran plot analysis (http://molprobity.biochem.duke.edu, accessed on 19 October 2022). It provides the number of residues that are located in the allowed, favored, and outlier regions. If a significant fraction of residues resides in the allowed and favored region, it is projected that the model is accurate [36].

## 3. Results

### 3.1. PLP1 Variants Associated with MS

PLP consists of 276 amino acid residues and four hydrophobic transmembrane domains, and its expression is restricted to oligodendrocyte cells. The area of the *PLP1* gene that encodes residue 31 appears to be a hotspot for mutation, since it has been described in MS patients (L31R and L31V mutation) [14]. In cases of severe PMD, the L31P mutation has also been documented [14]. The idea of mutation hotspots in *PLP1* has been previously characterized in PMD patients, and numerous mutations have been detected in a number of amino acids [37]. R137W mutation has also been described in MS patients, while the H140Y one was selected because it is the closest known mutation to residue 137 [14]. Detailed information about the variants analyzed in the current study is shown in Table 1. 

### 3.2. Variant Functional Analysis

There are numerous assessment strategies for missense variants and recent databases include results from a variety of techniques to assist the evaluation of the impact of variations predicted to modify the peptide sequence of a gene. Herein, using dbNSFP, we investigated the functional consequences of missense SNPs, including whether they are normal, disease-causing, or effective by chance. As Table 2 shows, functional analysis revealed that R137W, L31P, L31V, L31R are damaging from the most prediction tools with a high score, while the results for H140Y were different across the different methods.

We strengthened our analysis using further computational tools such as ANNOVAR, KGGSeq and VarSome and the outcomes were validated using each platform separately.

The results of MutPred2 demonstrated that these variants may alter the function of protein structures (Table S1). MutPred2 provides a general score which represents the average of all neural network scores based on a ranked list of specific molecular alterations potentially affecting the phenotype, and therefore, this number indicates the probability that the amino acid substitution could be harmful. A score threshold of 0.50, if considered as a probability, could reveal pathogenicity. However, a threshold of 0.68 results in a false positive rate (fpr) of 10%, whereas a threshold of 0.80 results in an fpr of 5%. In our case, L31P (score 0.973), L31V (score 0.854), and L31R (score 0.972) mutations may result in an altered transmembrane protein (Table S1). The R137W variant (score 0.684) may lead to a loss of helix, whereas the H140Y variant (score 0.556) may result in a changed ordered interface or transmembrane protein.

### 3.3. Conformational Analysis and Alteration of Protein Stability upon Amino Acid Substitution

**Figure 1:**
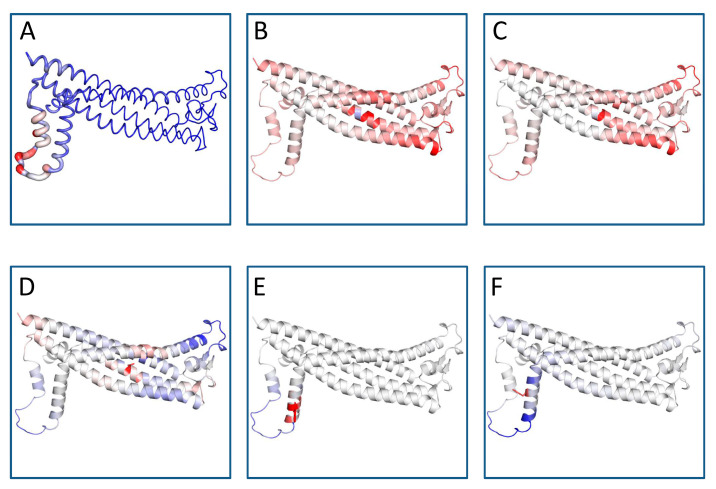
Protein flexible conformation based on the Vibrational Entropy difference (ΔΔS) between wild-type and mutant structures on the structure of PLP1. A visual representation of the chain in which the mutation occurs is also mapped. Amino acids colored according to the vibrational entropy change upon mutation. Blue represents a rigidification of the structure and red represents a gain in flexibility. (**A**) Normal PLP1; (**B**) L31P mutant; (**C**) L31R mutant; (**D**) L31V mutant; (**E**) R137W mutant; (**F**) H140Y mutant. The image is illustrated by DynaMut. The positions of the point mutations are 31, 137 and 140. Abbreviations: L is leucine; P is proline; R is arginine; V is valine; W is tryptophan; H is histidine; Y is tyrosine.

DynaMut predicts the change in stability by calculating the changes in unfolding Gibbs free energy (ΔΔG), as summarized in Table 3. For comparison, ΔΔG predictions based on protein structure were also displayed including dinstinct approaches and assumptions. Parallel analysis was performed using Site Directed Mutator (SDM) computational methods to verify the molecular effect of the five variants (Table S2). Three of them (L31V, L31P L31R) revealed a diminution in stability by increasing the molecular flexibility of the wild-type proteins (Table 3 and Table S2). On the contrary, H140Y variant enhanced the stability of the PLP1 protein. R137W variant revealed conflicting results. DynaMut demonstrated that the amino acid changes in R137W decrease stability, while SDM exhibited elevated stability (Table S2). ENCoM analysis was executed to calculate the vibrational entropy difference (ΔS) between wild-type and mutant structures as well as to explore protein conformational space and the effect of mutations on protein function and stability. As Figure 1 illustrates, the mutation causes a change in the vibrational entropy of the amino acid. 

### 3.4. Analysis of the Structural Conformation and Conservation of PLP1

**Figure 2:**
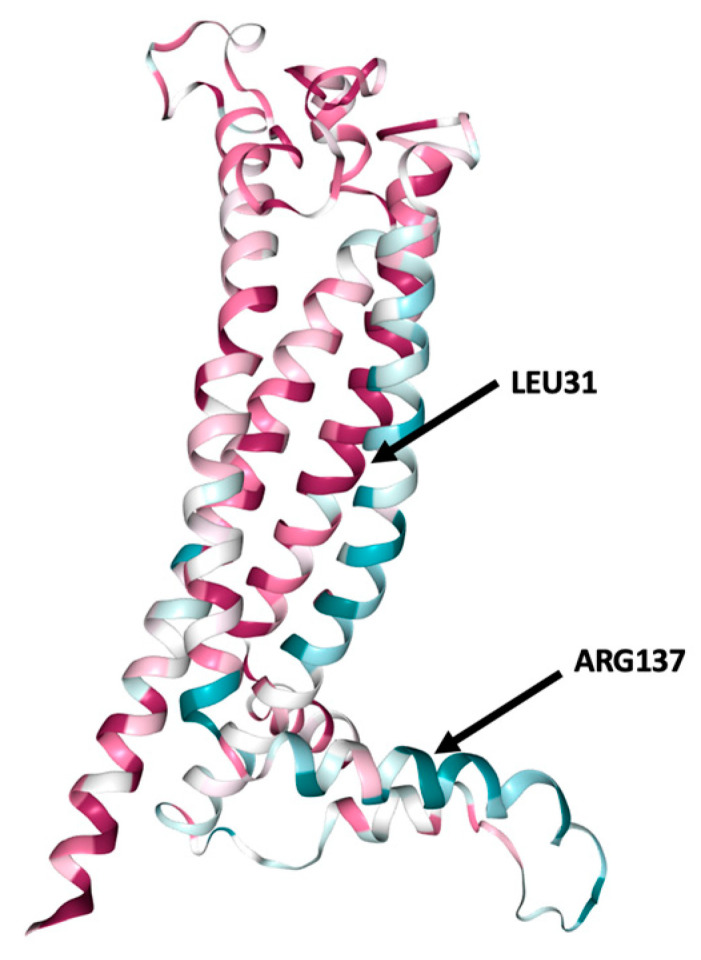
ConSurf analysis of conserved functional areas of the structural model of PLP1 gene-encoded protein. Amino acid at positions 31 (leucine) and 137 (arginine) are highlighted. Leucine in position 31 is a highly conserved region. Conservation score is presented in Figure S1.

According to ConSurf analysis, the variants located at position 31 (L31V, L31P, and L31R) were found in a highly conserved region with a conservation score of 9 (Figure 2). Based on this indication, we can estimate that these nsSNPs play a functional role on the protein conformation. On the contrary, R137 and H140 displayed a conservation score of 1 (Figure S1).

### 3.5. PLP1 Protein Secondary Structure Prediction

The alpha helix, beta sheet distribution and coils for PLP1 were calculated according to PSIPRED protein structure prediction server. Among the exposed secondary structures, the highest in percentage was alpha helix (65%) followed by coils (30%) and no beta-sheet (0.0%) (Figure S2).

### 3.6. Prediction Software Benchmarking and Creation of Tertiary Protein Structures

Using four distinct homology modeling techniques, the three-dimensional (3D) structures of the PLP1-encoded protein were reconstructed. Since only 3% of residues 45–53 is represented on the protein data bank (https://www.ebi.ac.uk/pdbe/pdbe-kb/, accessed on 10 October 2022), there was no known crystal data of this protein of the appropriate length. Once the mutations were identified, the next step was to construct protein-based structures to represent these variants. Since the 3D protein feature view was not determined through experimental methodologies, established computational tools and databases such as Uniprot (UniProt Consortium, London, UK, 2015), Swiss-Model, Phyre-2, I-TASSER, C-I-Tasser, PDBeFold and Dynamut were evaluated for predicting the mutated structures and calculated the effect of these domain mutations on the 3D protein structure. 

**Figure 3:**
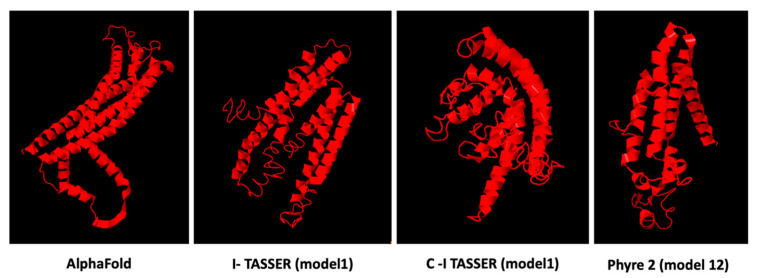
Protein structure prediction models of PLP1 calculated by different computational methodologies. AlphaFold model presented high confidence for the residue of the protein at position 31 (pLDDT > 90), but limited confidence for the PLP1 residue at position 137 (pLDDT < 50). Model 1 showed the highest C- score (−3.95) in I-TASSER and C-I-Tasser (−3.89) servers. Phyre2 model 12 with a confidence of 16.98% was the only one that included the residues analyzed in this study.

Based on the Hidden Markov approach, the Phyre-2 server was implemented to predict the homology-based three-dimensional structure of the query amino acid sequence. It incorporates five phases to construct a model: (1) collection of homologous sequences, (2) screening of fold library, (3) modeling of loops, (4) ab initio folding simulation Poing for multiple template modeling, and (5) placement of side chains [31]. Tertiary protein structures were formed based on the available structure prediction tools. Out of an extensive benchmarking of the structural predictive tools, we selected to retrieve the PLP1 target structure from the AlphaFold database. Comparison results revealed that AlphaFold reached the highest accuracy between predicted and experimental structure. In Figure 3, the visualization of the predicted 3D model of PLP1 protein is presented as performed by AlphaFold, Phyre-2, I-TASSER and C-I-Tasser, respectively, as these servers exhibited the highest accuracy in predicting the experimental structure. The model–template alignment by Swiss Model retrieved structures that did not include amino acids involved in the present outcomes.

### 3.7. Variant Tertiary Protein Structures 

**Figure 4:**
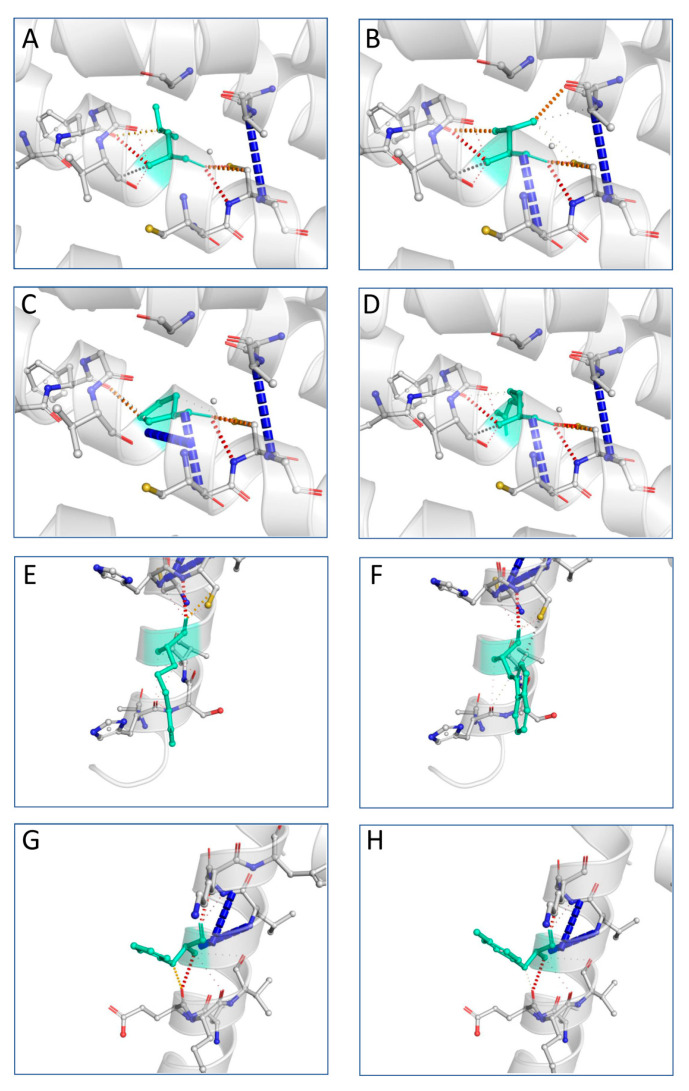
Interatomic interactions for wild-type and mutant PLP1. Both wild-type and mutant residues are colored in light green and depicted as sticks, along with domains participating in any interactions surrounding them. Leucine at position 31 is hydrophobic and highly conserved: (**A**) wild-type residue at position 31; (**B**) L31V (hydrophobic); (**C**) L31P (nonpolar); (**D**) L31R (polar). Arginine at position 137 and histidine at position 140 are polar: (**E**) wild-type residue at position 137; (**F**) R137W (aromatic); (**G**) wild-type residue at position 140; (**H**) H140Y (aromatic). The image is illustrated by DynaMut. A scale of color definition for each type of interaction is provided by software: red depicts hydrogen bonds; slight red depicts water-mediated hydrogen bonds; blue depicts halogen bonds; gold depicts ionic interactions; purple depicts metal complex interactions; light blue depicts aromatic contacts; green depicts hydrophobic contacts; pink depicts carbonyl contacts. The positions of the point mutations are 31, 137 and 140. Abbreviations: L is leucine; P is proline; R is arginine; V is valine; W is tryptophan; H is histidine; Y is tyrosine.

The next step in our pipeline was to construct the structures for these mutations using I-TASSER and DynaMut [26]. The DynaMut provides a comprehensive evaluation and visualization of protein mobility and flexibility using two independent, well-established normal mode approaches to analyze protein dynamics by sampling conformations. In parallel, assessment of the effect of mutations on protein dynamics and stability due to changes in vibrational entropy can be executed. The server combines graph-based signatures with normal mode dynamics to predict the influence of a selected mutation on protein stability. The predicted models were compared against the corresponding AphaFold structure through the TM-align algorithm and a benchmarking of the structural predictive tools was accomplished. Comparative results revealed that DynaMut reached the highest accuracy between the predicted and experimental structure and was also used to verify the impact of mutations on protein conformation, flexibility and stability as well as to visualize protein dynamics. The TM-score is the metric that will lead to the selection of the ideal approach for producing the potential tertiary structures of a protein. TM-align generates an optimal residue-to-residue alignment based on structural similarity utilizing dynamic programming iterations for two protein structures of uncertain equivalence [38]. TM-score for the five mutated structures shows that they were approximately in the same fold with the normal protein. Figure 4 presents the predicted interatomic interactions calculated for the wild-type protein and the single point mutations. Both wild-type and mutant residues are colored in light green and depicted as sticks, along with domains participating in any interactions surrounding them. 

### 3.8. Validation of the Predicted Structures

The Ramachandran plot was used to examine the conformation of the protein’s backbone. It represents an x-y plot of the phi/psi dihedral angles between NC-alpha and Calpha-C bonds. The Ramachandran plot of the wild-type protein in the AlphaFold model revealed 259 residues (94.2%) in the favored regions, 268 (97.5%) in the allowed regions and seven residues in the outlier region (Figure S3). The mutant protein structures obtained by DynaMut demonstrated the same results with wild-type PLP1. On the contrary, I-TASSER structure prediction models display poor Ramachandran plots compared to other algorithms. I-TASSER generates a model by reassembling structural parts from various templates, hence the model occasionally features unfavorable Ramachandran plot regions (Figure S4). The homology models indicated that PLP1 protein models obtained by DynaMut were accurate and they are useful for conducting additional studies and gaining a deeper understanding of the biological activity of the studied protein.

## 4. Discussion

In this study, the majority of tools indicated that MS-associated PLP1 mutations would have a significant impact on the protein structure, stability and function. Our analysis employed several computational approaches to predict the effects of the PLP1 gene variants, and important results were obtained. Examination of the modified protein structure revealed a destabilizing effect and an increase in flexibility. Loss of protein thermodynamic stability can reduce the ability of its structure to perform normal functions. Furthermore, precise analysis using MutPred2 revealed that these variants may affect protein functionality and structure. We used this machine learning-based approach to integrate data to reason probabilistically about the pathogenicity of amino acid substitutions. The resulted predictions for L31P, L31V and L31R indicated that these mutations may lead to an altered transmembrane protein. The R137W variant may also cause loss of helix, while the H140Y variant may alter the ordered interface of transmembrane protein. The findings of this study provide important insights for future investigations aimed at determining the role of PLP1 in MS.

Missense mutations have a substantial effect on protein functionality. A comprehensive computational examination of the phenotypic characteristics associated with specific variants can reveal the vulnerabilities that interfere with the normal protein activity. This study suggests that mutations in myelin-related genes may play a role in the development of MS. There are two putative PLP1-related MS mechanisms: PLP1 mutations could damage oligodendrocytes [39], generating an inside-out disease process, or they could cause the expression of neoantigens that the immune system could target [14]. Both can occur concurrently, so PLP1 should be investigated further. Previous studies showed that a wide variety of PLP1 genetic alterations have been identified as the underlying causes of PMD and SPG2 [40,41]. Understanding the pathophysiology of the disorders illustrated by a genotype–phenotype correlation requires an understanding of their cellular and metabolic impacts. The consequences of pathological modifications of PLP1 gene were better understood than the physiological functions of the PLP1 protein [37]. After more than 50 years of research, most of the intracellular mechanisms related to PLP1 functionality are still unknown, although the remarkable level of sequence conservation suggests that many mutations could cause severe implications, including MS [14]. 

In the present study, for most of the known variants, the 3D structures of the proteins are not experimentally known, so there is a clear lack of experimental evaluations of variant effects. Prediction methods can help close the sequence-annotation gap, but with respect to deep annotations of function, in silico methods remain limited. These methods are mainly oriented towards intrinsically disordered proteins and clustered data are based on sequence identity thresholds, retaining a single representative sequence from each group. This approach results in models that resemble having learned a concept instead of a probability distribution. Well-defined theoretical support for this situation is an open problem that will formalize and improve understanding of this long-standing practice in computational biology. 

MS is a persistent autoimmune inflammatory disease of the human central nervous system (CNS). It is characterized by loss of motor and sensory function resulting from immune-mediated inflammation, demyelination and sequelae destruction of nerve axons. Along the axon, there are intermittent points that are not surrounded by myelin and are called junctions of Ranvier [42]. MS shows great diversity both at the point of disease onset and at the stage of developmental progression. Four main types of the disease are distinguished: Relapsing–Remitting MS (RRMS) that is characterized by clearly defined relapses of increased disease activity and the worsening of symptoms; Secondary Progressive MS (SPMS), the next step of the RRMS progress for the majority of patients; Primary Progressive MS (PPMS), presenting with symptoms that have been steadily worsening since onset of the disease, without relapses or remissions; and finally Progressive Relapsing MS (PRMS) that is progressive from onset with continuous worsening between relapses [43,44]. Myelin proteins are considered potential targets of the immune system in MS, and activated T-cells recognize specific myelin epitopes at sites of extensive demyelination. According to clinical, pathologic, imaging and electrophysiologic studies, it is not yet understood whether MS is beginning in the periphery, by stimulation of immune cells that thereafter penetrate the CNS and cause damage, or within the CNS through primary myelin or oligodendrocyte injury [45]. This could be the result of mutations in molecules encoding critical components of myelin or oligodendrocytes despite the fact that genome-wide association studies have not found significant links between them and multiple sclerosis. However, it remains possible that mutations in genes encoding these components may be present in a subset of MS patients. In this regard, missense mutations in PLP1 have been identified in patients exhibiting clinical symptoms consistent with a diagnosis of multiple sclerosis [14]. 

Although the pathogenesis of MS remains unclear, multiple genes, generally of poor penetrance, have been related to MS susceptibility, and their nature suggests autoimmunity causes disease development in most cases [46]. MS is a serious autoimmune disease, unfortunately without a cure; however, over the last three decades, there has been a rapid expansion of therapeutic approaches for the disorder including immunoprotective strategies, shingosine-1-phoshate receptor modulators and cell-based therapies [47]. Emphasis should be placed on early identification of risk factors for early therapeutic interventions. The disease has a different pathogenetic factor in each patient. PLP1 mutations L31V, L31R and R137W could impair PLP trafficking out of the ER and induce the unfolded protein response (UPR). The data imply that PLP1 mutations could have a harmful effect on oligodendrocyte functionality and consequently cause MS [14]. This is confirmed by recent finding in mice carrying the L31R and R137W mutations: they showed neuroinflammation, axonal degeneration, neuronal loss, and brain shrinkage by one year [18]. The same mutations and the loss of function of glial *PLP1* gene indicated a clinical scenario similar to MS in humans. The area of PLP1 gene encoding residue 31 appears to be a hotspot for mutation as L31P has been linked to severe PMD. The L31V mutation shows the least effects on PLP expression, trafficking, or UPR induction is a conservative mutation, as we already stressed [14]. It is not expected to have a significant impact on the hydrophobicity of the first transmembrane region in which it is located, as L and V are hydrophobic amino acids with similar structures and neutral side chains. An L31R mutation in the first transmembrane domain of PLP could affect the overall charge, hydrophobicity, and/or secondary structure of the transmembrane helix, disrupting PLP structure. The L31P mutation would force a stiff bend on the polypeptide and damage the transmembrane helix. R137W occurs in exon 3B, which is deleted in PLP DM20. DM20 is expressed before PLP during ontogenesis and may play a role in the development of new oligodendrocytes [48]. Several L31V-mutated peptides were expected to bind with higher affinity to some of the patient’s HLA molecules than the native peptide, producing de novo epitopes and potentially inducing/activating a new group of autoreactive T cells [14,49]. Such responses depend on the presence of proteases that can digest peptides and T cells that can recognize novel epitopes in the patient’s T cell repertoire.

## 5. Conclusions

PLP1 plays an important role in myelin structure and stability, an insulating lipoprotein which helps transmit nerve impulses. Numerous computational tools were utilized in the present in silico analysis, which demonstrated that the amino acid changes L31V, L31R, and R137W of the PLP1 protein are functionally detrimental. The L31V and L31R variants of PLP1 reside in the conserved domain of the protein. To examine the stability of mutant and wild-type PLP1 proteins, we also calculated the changes in their free energies. Our findings provide evidence for the functional role of these three variations, which facilitates the establishment of accurate insights for drug targeting and future clinical application in patients with multiple sclerosis. Alteration of overall cellular activity often arises as a consequence of altered function of one or more individual proteins. Identification of more variants as specific targets may provide a better understanding of conformational dynamics for future studies, while molecular recognition specific to mutated proteins will play an important role in broadening the scope of intracellular mechanisms involved in inflammatory demyelinating diseases.

### Key Points

1. The majority of computational tools indicated that MS-associated *PLP1* mutations would have a significant impact on the protein structure, stability and function.
2. Loss of protein thermodynamic stability can reduce the ability of its structure to perform normal functions.
3. The resulted predictions for L31P, L31V and L31R indicated that these mutations may lead to an altered transmembrane protein.
4. The R137W variant may also cause loss of helix, while the H140Y variant may alter the ordered interface of transmembrane protein.

## Figures and Tables

**Table 1:** PLP1 variants. This mutation was selected as the closest mutation to residue 137 known for *PLP1*.

Location	Codon Change	Amino Acid Position	Amino Acid Alteration	Description ^1^
X:103785668	CTG/GTG	31	L/V	MS- like disease mutation
X:103785669	CTG/CCG	31	L/P	Severe PMD mutation
X:103785669	CTG/CGG	31	L/R	MS- like disease mutation
X 103786682	CGG/TGG	137	R/W	MS- like disease mutation
X 103786691	CAT/TAT	140	H/Y	Mild SPG2 mutation

^1^ MS: multiple sclerosis; PLP: myelin proteolipid protein; PMD: Pelizaeus–Merzbacher disease; SPG2: spastic paraplegia type 2.

**Table 2:** Functional analysis of PLP variants using dbNSFP.

Variant	L31P	L31V	L31R	R137W	H140Y	Range (Low to Damaging)
Polyphen2_HDIV_score	0.999	0.997	0.999	0.999	0.015	0.03061 to 0.91137
LRT_converted_rankscore	0.8433	0.8433	0.8433	0.4496	0.53742	0.00162 to 0.8433
MutationTaster_score	1	0.999999	1	0.627105	0.281663	0 to 1
MutationTaster_converted_rankscore	0.81001	0.58761	0.81001	0.81001	0.81001	0.08979 to 0.81001
MutationAssessor_score	2.71	2.36	2.71	1.24	0.69	−5.17 to 6.49
MetaLR_score	0.9794	0.9771	0.9794	0.9547	0.9047	0 to 1
MetaRNN_score	0.988245	0.946438	0.98141	0.892939	0.962404	0 to 1
MutPred_score	0.932	0.813	0.887	0.663	0.779	0 to 1
DEOGEN2_score	0.994664	0.910984	0.994756	0.707527	0.697598	0 to 1
ClinPred_score	0.996376	0.982601	0.997494	0.958003	0.509568	0 to 1

**Table 3:** Conformational Analysis of Protein’s Stability Change upon Amino Acid Substitution.

Variant	ΔΔG (kcal/mol)	Outcome	ΔΔSVibENCoM (kcal.mol^−1^.K^−1^)	Outcome ^1^
L31V	−0.133	Destabilizing	0.083	Increase
L31P	−1.011	Destabilizing	0.413	Increase
L31R	−0.256	Destabilizing	0.231	Increase
R137W	−0.400	Destabilizing	0.111	Increase
H140Y	0.519	Stabilizing	−0.063	Decrease

^1^ Molecule flexibility.

## Data Availability

Not applicable.

## Supplementary Materials

The following supporting information can be downloaded at: https://www.mdpi.com/article/10.3390/brainsci13010042/s1, Table S1: Prediction of pathogenicity of amino acid substitutions of *PLP1* variants; Table S2: Analysis of missense mutations on protein stability; Figure S1: Prediction of evolutionary conserved amino acid residues of PLP1; Figure S2: Protein secondary structure predictions of PLP1; Figure S3 and Figure S4: Ramachandran plots.
