# Supplementary material for: In Silico Structural Analysis Predicting the Pathogenicity of PLP1 Mutations in Multiple Sclerosis
Source: Brain Sci. 2022 Dec 24;13(1):42. doi: 10.3390/brainsci13010042 (PMC9856082; doi:10.3390/brainsci13010042)
Supplement: Supplementary file 1 [file brainsci-13-00042-s001.zip › brainsci-2105939-supplementary.pdf]

Supplementary Material

In Silico Structural Analysis Predicting the Pathogenicity of PLP1 Mutations in Multiple Sclerosis

Antigoni Avramouli, Marios G. Krokidis *, Themis P. Exarchos and Panagiotis Vlamos

Bioinformatics and Human Electrophysiology Laboratory, Department of Informatics, Ionian University, 491 00 Corfu, Greece

*Correspondence: mkrokidis@ionio.gr

Table S1. Prediction of pathogenicity of amino acid substitutions and their molecular mechanisms of *PLP1* variants provided by MutPred2

Variant	MutPred2 Score	Molecular mechanism disrupted	Probability	P-value
L31P	0.973	Altered Transmembrane protein	0.33	6.8e-05
L31V	0.854	Altered Transmembrane protein	0.26	1.2e-03
L31R	0.972	Altered Transmembrane protein	0.33	4.9e-05
R137W	0.684	Loss of Helix	0.31	3.1e-03
H140Y	0.556	Altered Ordered interface	0.31	2.3e-03,
		Altered Transmembrane protein	0.11	0.04

Table S2. Analysis of missense mutations on protein stability, performed by SDM and DynaMut. DynaMut data (Table 3) are included for comparison.

	DynaMut				SDM[1]	
**Variant**	**ΔΔG (kcal/mol)**	**Outcome**	**ΔΔSVibENCoM (kcal.mol^{-1}.K^{-1})**	**Outcome**	**ΔΔG (kcal/mol)**	**Outcome**
L31V	-0.133	destabilizing	0.083	increase	-0.67	destabilizing
L31P	-1.011	destabilizing	0.413	increase	-2. 23	destabilizing
L31R	-0.256	destabilizing	0.231	increase	-0.29	destabilizing
R137W	-0.400	destabilizing	0.111	increase	0.25	stabilizing
H140Y	0.519	stabilizing	-0.063	decrease	0.65	stabilizing

[1]Site Directed Mutator

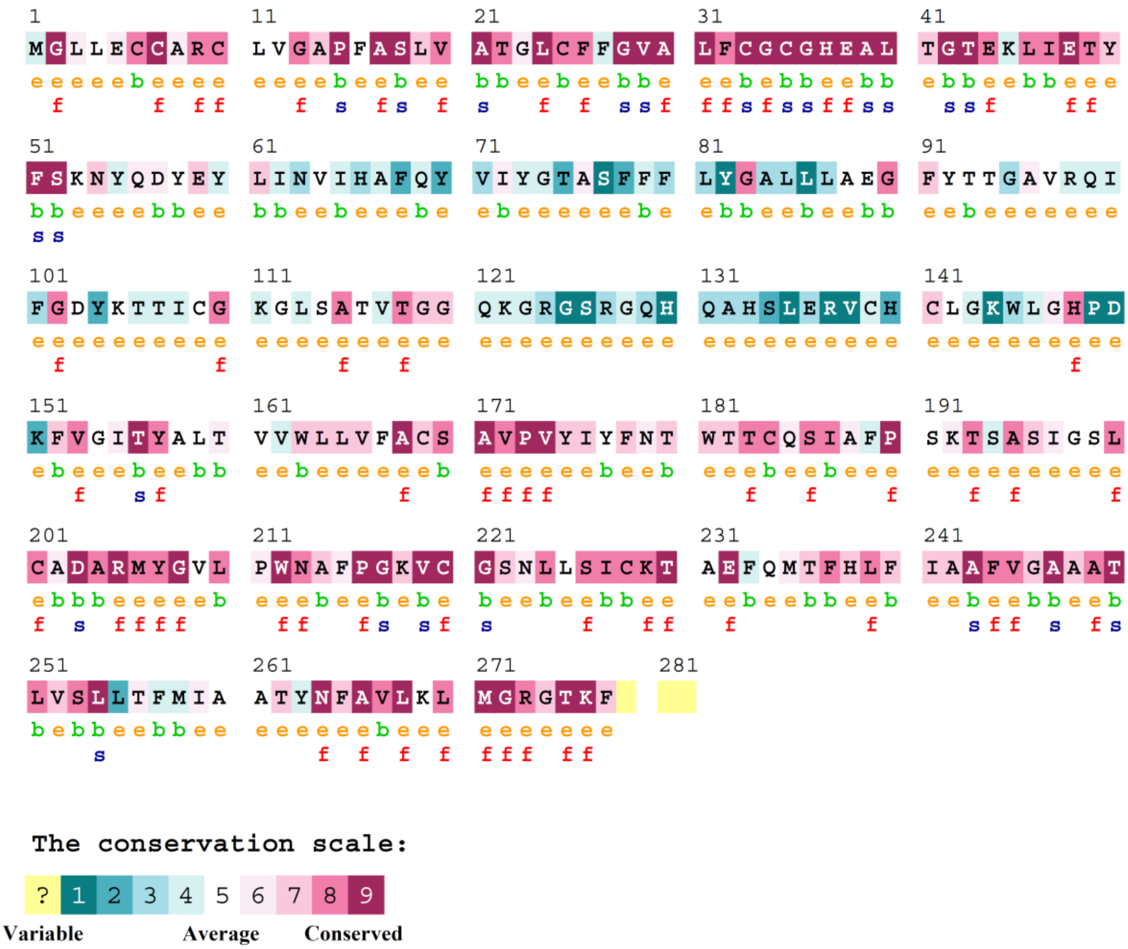

Figure S1. Prediction of evolutionary conserved amino acid residues of PLP1, performed by ConSurf server. Conservation score is represented as color coding bars.

e: an exposed residue according to the NACSES algorithm; b: a buried residue according to the NACSES algorithm; f: a predicted functional residue (highly conserved and exposed); s: a predicted structural residue (highly conserved and buried).

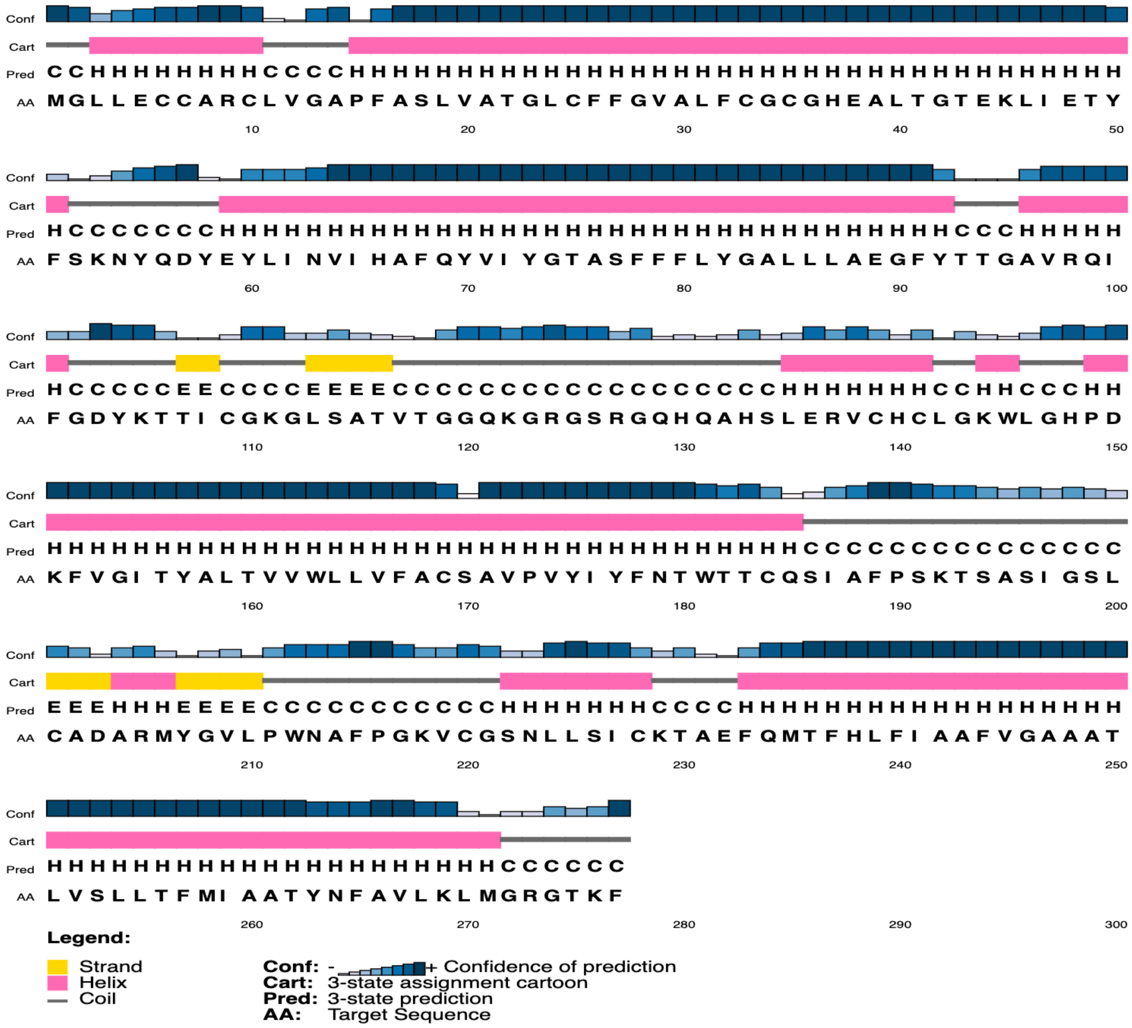

Figure S2. Protein secondary structure predictions of PLP1 by PSIPRED tool. The graphical output of PSIPRED prediction of the secondary structure of the protein shows 10 α-helices extends from 3th to 10th, 15th to 51th, 59th to 92th, 96th to 101th, 135th to 141th, 144th to 146th, 149th to 185th, 204th to 206th, 222th to 228th and 233th to 271th residue and no β-strands.

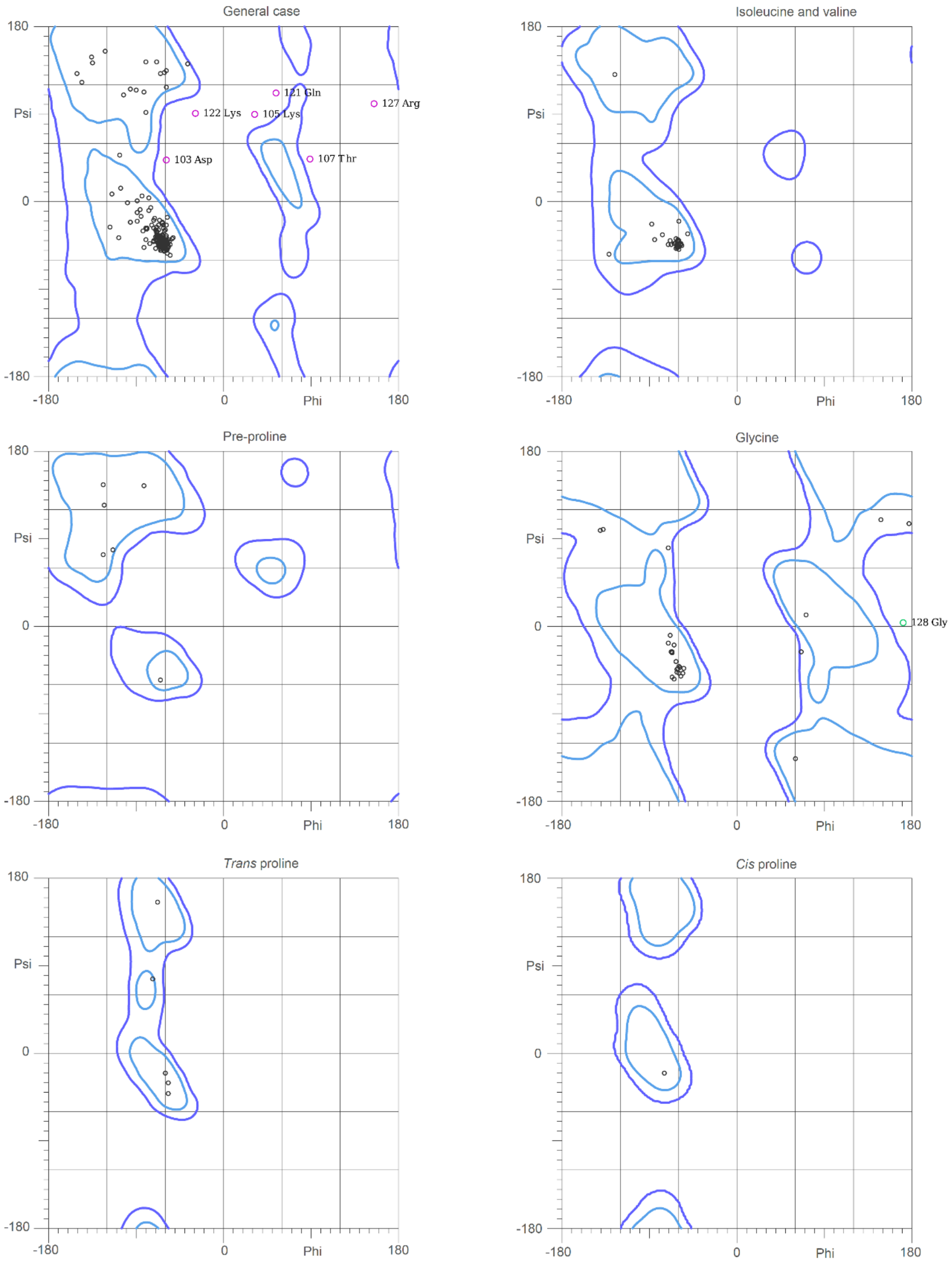

Figure S3. Ramachandran plots of PLP1 in the AlphaFold model.

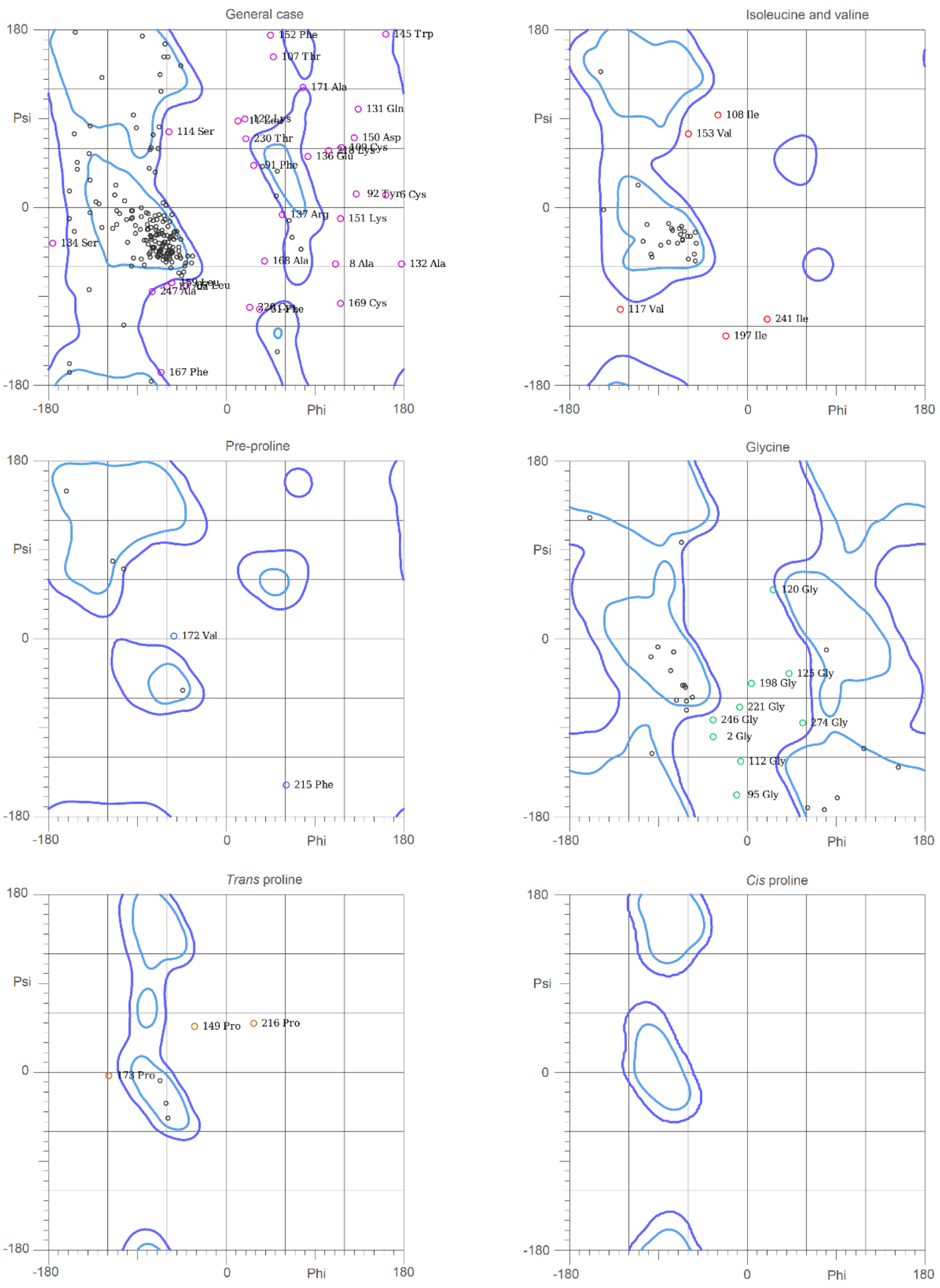

Figure S4. Ramachandran plots of PLP1 in the I-Tasser model.
